# Differential transcriptome dynamics during the onset of conceptus elongation and between female and male porcine embryos

**DOI:** 10.1186/s12864-019-6044-z

**Published:** 2019-08-28

**Authors:** Shuqin Zeng, Jochen Bick, David Kradolfer, Johannes Knubben, Veronika L. Flöter, Stefan Bauersachs, Susanne E. Ulbrich

**Affiliations:** 1 ETH Zurich, Animal Physiology, Institute of Agricultural Sciences, Zurich, Switzerland; 2 University of Zurich, Genetics and Functional Genomics, Clinic of Reproductive Medicine, Department for Farm Animals, Zurich, Switzerland; 3 Physiology Weihenstephan, Technical University Munich, Freising, Germany

**Keywords:** Pig, Embryo, X-linked gene, Transcriptomics, Preimplantation, RNA-Seq

## Abstract

**Background:**

Porcine embryos undergo rapid differentiation and expansion between Days 8 and 12 before attaching to the maternal uterine epithelial surface after Day 13. It is known that maternal recognition of pregnancy and successful implantation are driven by mutual interactions between the elongated conceptus and the maternal endometrium. While most of the genes involved in regulation of embryo development are located on autosomal chromosomes, gene expression on sex chromosomes is modulating development through sex-specific transcription. To gain more insights into the dynamic transcriptome of preimplantation embryos at the onset of elongation and into X-linked gene expression, RNA-seq analyses were performed for single female and male porcine embryos collected on Days 8, 10, and 12 of pregnancy.

**Results:**

A high number of genes were differentially expressed across the developmental stages (2174 and 3275 for Days 8 vs 10, and 10 vs 12, respectively). The majority of differentially expressed genes (DEGs) were involved in embryo elongation, development, and embryo-maternal interaction. Interestingly, a number of DEGs was found with respect to embryo sex (137, 37, and 56 on Days 8, 10 and 12, respectively). At Day 8, most of these DEGs were X-linked (96). Strikingly, the number of DEGs encoded on the X chromosome dramatically decreased from Day 10 to Day 12.

**Conclusions:**

The obtained results deepen the understanding about temporary transcriptomic changes in porcine embryos during the phase of conceptus elongation, meanwhile reveal dynamic compensation of X chromosome in the female and distinct transcriptional differences between female and male embryos.

**Electronic supplementary material:**

The online version of this article (10.1186/s12864-019-6044-z) contains supplementary material, which is available to authorized users.

## Background

Porcine embryos undergo a morphological transformation before attaching to the uterine surface, which is supposed to maximize the intimate embryo-maternal communication. This is essential for maternal recognition of pregnancy and proper embryonic development during the preimplantation period, and thus a prerequisite for a successful pregnancy [1]. The transformation of morphology is likewise essential for embryo survival as the increased contact surface area improves the nutrient exchange between the conceptus and the uterus [2]. Embryos in the morula stage are transported into the anterior tips of the uterine horns around 60–72 h after estrus, thereafter developing into blastocysts until Day 5 of pregnancy. The blastocysts start hatching around Day 8 [3]. After hatching, porcine embryos continue to grow and form a sphere of around 2 to 6 mm in diameter on Day 10. Finally, the spherical blastocysts are transformed to tubular and then a long and thin filament approximately 100–150 mm in length on Day 12 of pregnancy [4]. Unlike the ruminant trophoblast-growth-driven elongation, the elongation from spherical to filamentous form of the pig conceptus is mainly contributed by comprehensive cellular migration and reorganization [5, 6].

Along with embryo elongation, estrogens secreted from the conceptuses on Days 11 and 12 initiate the most pronounced phase of maternal recognition of pregnancy [7, 8]. A number of genes from the uterine epithelium mediating cell growth, adhesion, transcription, transport, as well as prostaglandins, amino acids, and glucose synthesis are stimulated by estrogens [8, 9]. Besides, the cellular signaling pathways and their corresponding receptors located in the conceptuses were found to play a role in modulating cell proliferation, movement, adhesion, and trophectoderm cells survival during preimplantation and implantation periods [3]. The developing conceptus also contributes to the accumulation of prostaglandin F2α (PGF) and prostaglandin E_2_ (PGE) in the uterine luminal content [5, 10, 11]. According to previous studies, porcine conceptus interleukin one beta (*IL1B*) mRNA and protein abundance is increasing during the transformation from the tubular to the filamentous stage, reaching a maximum at the highly elongating period [12]. It is known that IL1B binds to a large number of receptors and its antagonists, together with an accessory protein, thereby regulating innate immunity and inflammation [13, 14]. Furthermore, interleukin 1 receptor (IL1R1) in the endometrium activated by conceptus IL1B2 triggers a cellular signaling pathway cascade through extracellular signal-regulated kinase 1/2 (ERK1/2), mitogen activated kinase-like protein (MAPK) [15] and nuclear factors kappa-B (NFKB) [16]. Then, the NFKB activation mediates numerous biological networks including cytokines, chemokines, and prostaglandin-endoperoxide synthase 2 (*PTGS2*) expression [17]. However, the dynamic changes of gene expression during the preimplantation period in the conceptus are still poorly understood.

Genes located on the sex chromosomes can modulate the genome machinery resulting in differences in the development of female and male embryos. However, the sole determinant in phenotypic differences is different chromosome dosages during embryo preimplantation development [18]. In females, most genes on one X chromosome are silenced because of X-chromosome inactivation (XCI) during early development [19]. As the model of dosage compensation during preimplantation development is proved by previous studies, a reversible dynamic X inactivation may cause X-linked gene up-regulation in female embryos [20]. The molecular events that cause differences between male and female embryos were observed in in vitro culture experiments including embryo developmental speed, blastocyst cell number, and metabolism [21]. In a global gene expression study of bovine Day 7 embryos, 193 X-linked transcripts were upregulated in female compared to male embryos, suggesting that XCI is partially achieved at the blastocyst stage [21]. To date, this is not clear in porcine embryos.

Considering the limited knowledge in pigs in terms of transcriptome changes of preimplantation embryos during the onset of elongation and respective differences between female and male embryos, this study was designed to analyze global gene expression in individual porcine embryos during the preimplantation period (Day 8, 10, and 12 of pregnancy) with the aims of (i) characterizing the dynamics of gene expression profiles and the involved functions and thereby (ii) investigating X-linked gene expression between female and male embryos.

## Results

### RNA sequencing of porcine embryo samples

**Fig. 1:**
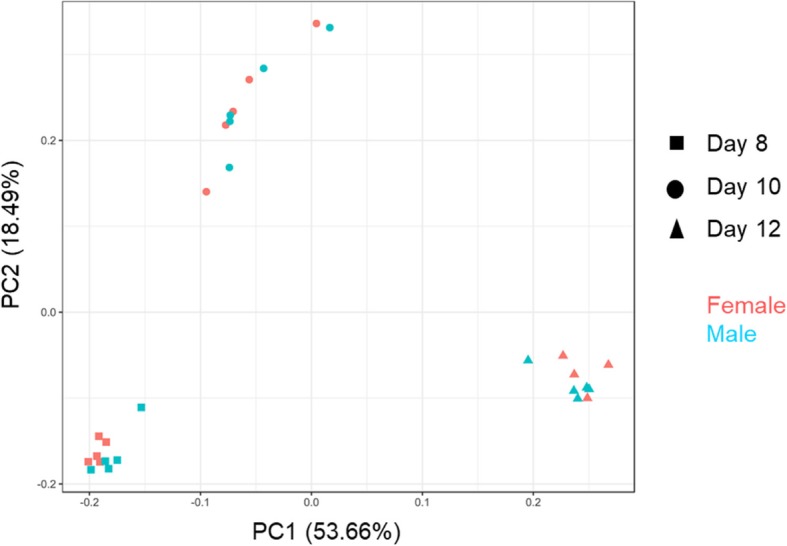
Principal component analysis of all genes expression patterns of embryos. The multidimensional scaling plot displays that embryos from the same stage are grouped together. Samples from the same stage with the same symbol (square, round, triangle shape indicate samples of Day 8, 10, and 12, respectively). Red and blue colors indicate female and male embryos, respectively

At least two embryos (one female and one male) were selected from the same sow in each stage and they were mixed within the same treatment. The embryos size collected on Days 10 and 12 ranged from 2.2 to 3 mm, and from 45 to 200 mm, respectively. RNA-seq libraries were prepared from 30 individual embryos (5 female and 5 male embryos each on Days 8, 10, and 12). The number of raw reads (100-bp single-end reads) ranged from 19 to 41 million per sample. From these, 18 to 39 million clean reads were obtained after quality filtering and adapter clip. All data used in this study have been included in the article and its Additional files. The sequence data (GSE113366) is available at National Center for Biotechnology Information (NCBI) Gene Expression Omnibus. The principal component analysis for all detected genes (13103) in these embryos (Fig. 1) revealed that embryos with the same days of pregnancy were grouped together. It was notable that embryo from Day 12 showed a higher distance with embryos from Days 8 and 10, besides the variation between embryos on Day 10 was highest compared with the rest embryos.

### Differentially expressed genes across the stages during the preimplantation phase

**Fig. 2:**
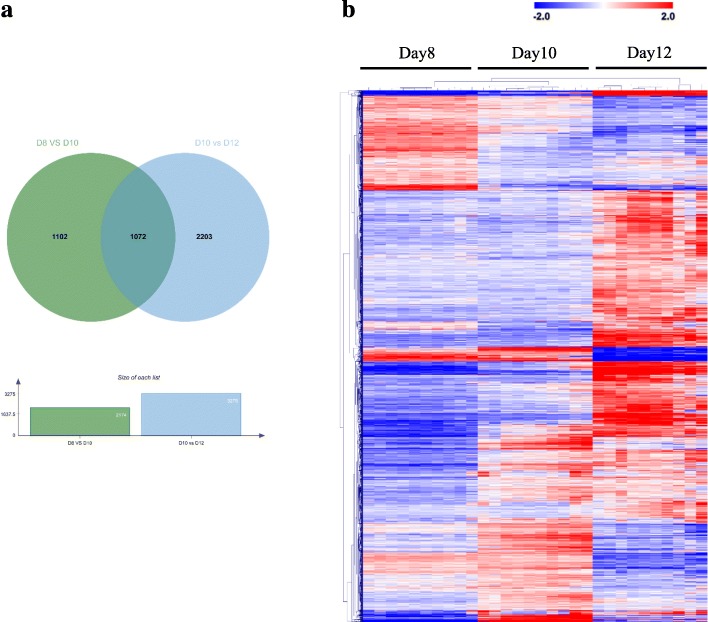
Venn diagram for differentially expressed genes identified across the three developmental stages (**a**). Green, Day 8 vs Day 10 of embryos; blue, Day 10 vs Day 12 of embryos. Hierarchical cluster analysis of differentially expressed genes identified for three stages (**b**). Mean-centered expression values (log2 counts per million of sample – mean of log2 counts per million of all samples) for the embryos of the Day 8 vs Day 10 and Day 10 vs Day 12 are shown for genes with significant differences in gene expression (FDR < 0.1% and │log2 fold change│ > 1). The color scale is from −2 (blue, lower than mean) to 2 (red, higher than mean). Each row represents 1 gene, each column 1 sample

**Fig. 3:**
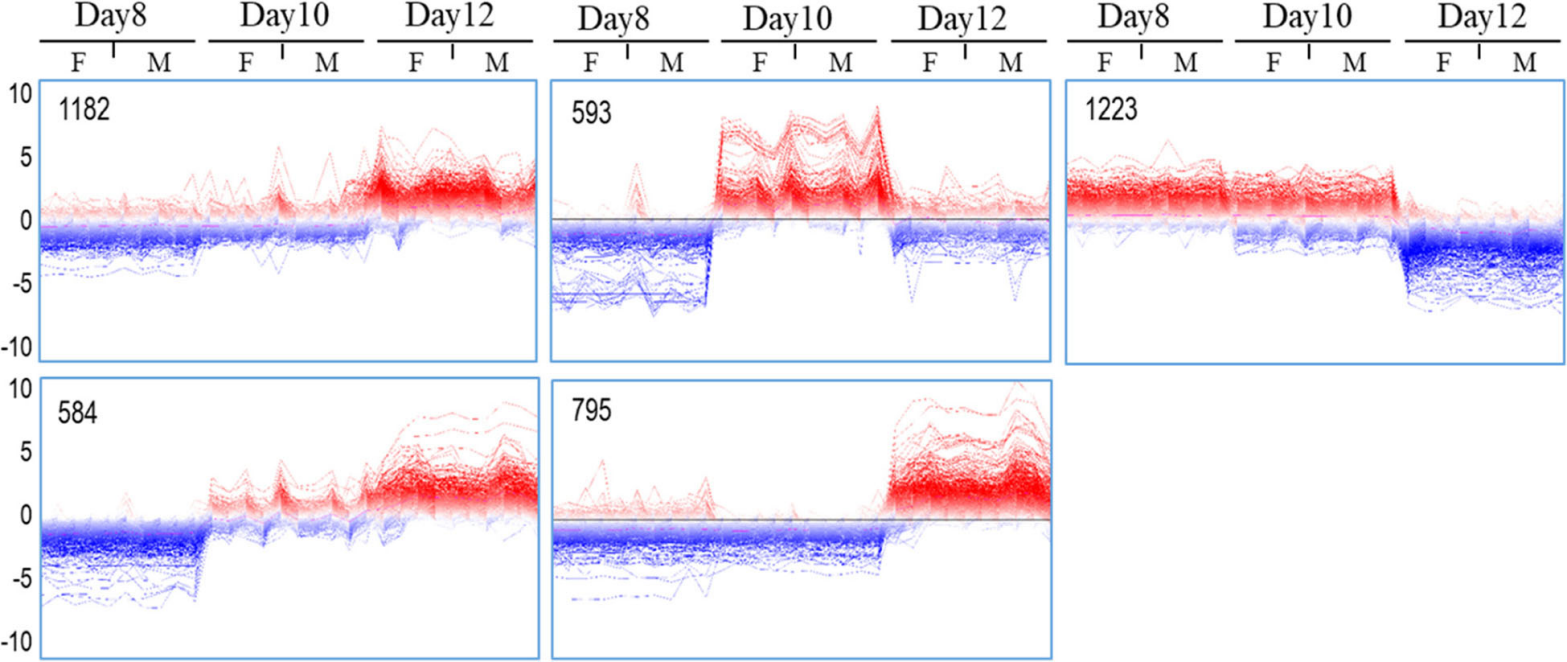
Clustering of gene expression profiles for three stages of the embryos. The SOTA of MeV software (version 4.9.0) was used to obtain groups of genes with similar expression profiles. Clusters 1 to 5 are from left to right and top to down. Vertical axis is in log2 scale and shows the deviation from the mean expression value. Numbers of genes for each cluster are shown (top left corner). F and M mean female and male embryos, respectively

Based on the analysis of DEG (FDR 0.1%, log2 fold change > 1), the total number of 2174 DEG were found between Days 8 and 10, and even more genes (3275 DEG) were differentially expressed between Days 10 and 12 (Fig. 2a). A number of 1072 DEG were shared by these two comparisons and many of them shared a similar expression profile. The DEG of each comparison were combined (4377 DEG) for analysis of typical expression profiles across the developmental stages. Hierarchical cluster analysis provided an overview of the expression profiles for all the combined DEG (Fig. 2b). To have a closer look into the regulation of these DEGs, the genes with similar expressions at the same stage were identified by Self-Organizing Tree Algorithm (SOTA) analysis (Fig. 3), which revealed the expression of almost half of the genes was similar on Days 8 and 10 but increased on Day 12 (genes of cluster 1 and 5). Inversely, a number of genes showed increased expression on Days 8 and 10 but decreased expression on Day 12 (cluster 3). Furthermore, clusters of genes were found that showed increased expression only on Day 8 (contained in cluster 3) and only on Day 10 (cluster 2), respectively. In cluster 4, the gene expression dramatically increased on Day 10, and kept a stable upregulated expression level on Day 12. The DEGs across the three stages are shown in Additional file 1: Table S1.

### Functional category analysis of gene expression profile clusters

A functional annotation clustering analysis was performed with the Database for Annotation, Visualization, and Integrated Discovery (DAVID) tool for the individual SOTA clusters. Highly significant functional annotation clusters (geometric mean of member categories’ *P*-values < 0.001 which was calculated based on Fisher’s exact test) for each of the 5 SOTA expression profile clusters are shown in Table 1. Strong enrichment of functional categories related to cell communication, cell motility and migration, cell adhesion and junction, cytoskeleton organization, embryo development, apoptosis, and immune response was found for genes specifically upregulated on Day 12 (clusters 1 and 5). For genes up-regulated genes on Day 10 (cluster 2), lipid biosynthetic and metabolic process, transport, developmental and homeostasis were enriched. Genes upregulated on Days 8 and 10 (cluster 3) revealed overrepresented functional categories such as vacuole, lipid metabolic and biosynthetic process, cell-cell junction, and ion transport. For cluster 4, containing genes with increased expression on Days 10 and 12 functional categories involved in development and morphogenesis, cell-cell signaling, and apoptotic process were overrepresented. More detailed information is shown in Additional file 2: Table S2.

**Table 1:** Overrepresented DAVID annotation clusters for the obtained SOTA expression clusters

Expression cluster	Representative enriched functional terms^a^	Enrichment Score^b^
Cluster1	extracellular region part (308, 1.39); extracellular vesicle (226, 1.4); extracellular exosome (224, 1.39)	8.8
	response to organic substance (225, 1.46); response to endogenous stimulus (124, 1.47)	7.77
	cell motility (134, 1.85); cell migration (123, 1.9)	7.71
	cell proliferation (159, 1.57)	7.57
	regulation of cell communication (244, 1.48); regulation of signal transduction (225, 1.51)	7.56
	circulatory system development (97, 1.88); angiogenesis (50, 2.22)	7.34
	regulation of multicellular organismal development (150, 1.6); positive regulation of developmental process (90, 1.52)	7.21
	response to cytokine (81, 1.85); cellular response to cytokine stimulus (71, 1.84)	6.22
	cell adhesion (142, 1.53); biological adhesion (142, 1.53)	5.11
	inflammatory response (64, 1.84); defense response (118, 1.43)	4.85
	peptidase regulator activity (32, 2.7); enzyme inhibitor activity (41, 1.95); regulation of proteolysis (62, 1.61)	4.68
	membrane region (43, 2.13); membrane raft (34, 2.15)	4.62
	multicellular organism metabolic process (23, 3.17)	4.37
	regulation of epithelial cell proliferation (35, 2.14); epithelial cell proliferation (37, 1.96)	3.69
	actin filament-based process (64, 1.75)	3.61
	lipid metabolic process (102, 1.41)	2.76
	response to oxygen-containing compound (113, 1.41)	2.12
Cluster2	lipid metabolic process (84, 2.47); lipid biosynthetic process (47, 2.95)	11.91
	cardiovascular system development (49, 2.03)	4.39
	chemical homeostasis (58, 2.17); homeostatic process (72, 1.71)	3.75
	ion transport (68, 1.83); cation transport (45, 1.81); transmembrane transport (56, 1.66)	3.37
	membrane depolarization (11, 4.74); regulation of membrane potential (23, 2.5)	3.31
	small molecule catabolic process (24, 2.76); carboxylic acid catabolic process (16, 3.23)	3.24
	movement of cell or subcellular component (73, 1.59); regulation of cell migration (34, 1.94)	2.87
	phospholipid metabolic process (27, 2.69); glycerophospholipid metabolic process (19, 2.68)	2.72
	intracellular signal transduction (98, 1.44); regulation of cell communication (107, 1.39)	2.59
	positive regulation of multicellular organismal process (59, 1.62)	2.57
	response to oxygen-containing compound (59, 1.58)	2.52
	phosphorus metabolic process (111, 1.41); phosphate-containing compound metabolic process (110, 1.4)	1.65
Cluster3	vacuole (131, 1.79)	8.09
	lipid metabolic process (118, 1.56); lipid biosynthetic process (57, 1.61)	5.41
	cell-cell junction (66, 1.7); protein binding involved in cell adhesion (37, 2.09); cadherin binding (35, 1.97); adherens junction (65, 1.55)	4.01
	sphingolipid metabolic process (26, 2.95); membrane lipid metabolic process (29, 2.5)	3.93
	extracellular organelle (217, 1.27); extracellular vesicle (216, 1.27); extracellular exosome (215, 1.27)	3.44
	pigment cell differentiation (11, 5.38); developmental pigmentation (11, 4.12)	3.28
	ion transport (123, 1.48); cation transport (88, 1.59); transmembrane transport (112, 1.49)	3.11
	biological adhesion (133, 1.37); cell adhesion (132, 1.37)	2.61
	cellular homeostasis (79, 1.67); homeostatic process (132, 1.41)	2.6
	embryonic organ development (43, 1.81)	2.38
	macromolecule localization (205, 1.28)	2.24
	actin filament-based process (61, 1.6)	2.07
	cellular response to oxygen-containing compound (79, 1.52)	2.03
	regulation of kinase activity (71, 1.59); phosphorus metabolic process (217, 1.24)	1.8
	regulation of molecular function (205, 1.26)	1.62
	regulation of cellular localization (72, 1.52)	1.43
Cluster4	epithelium development (57, 2.03); organ morphogenesis (48, 1.83)	4.55
	urogenital system development (27, 3.19)	3.81
	blood vessel development (34, 2.23); vasculature development (35, 2.17); cardiovascular system development (47, 1.88); circulatory system development (47, 1.88)	3.73
	extracellular exosome (113, 1.42); extracellular vesicle (113, 1.41); extracellular organelle (113, 1.41); extracellular region part (141, 1.28)	3.7
	cell surface receptor signaling pathway involved in cell-cell signaling (32, 2.29); Wnt signaling pathway (29, 2.34); cell-cell signaling by wnt (29, 2.33)	3.61
	regulation of signal transduction (101, 1.4)	3.4
	apoptotic process (76, 1.64); cell death (80, 1.54)	3.4
	reproductive system development (36, 3.21)	3.28
	actin filament-based process (37, 2.08); actin cytoskeleton organization (32, 2.18)	2.77
	regulation of hydrolase activity (61, 1.72); molecular function regulator (62, 1.64); enzyme regulator activity (46, 1.7)	2.68
	movement of cell or subcellular component (74, 1.56); regulation of cell motility (37, 1.9)	2.46
	regulation of signaling (112, 1.38); regulation of cell communication (110, 1.38)	2.31
	regulation of cell proliferation (65, 1.58)	2.26
	nervous system development (84, 1.46); negative regulation of cell differentiation (33, 1.95)	1.72
Cluster5	cell death (124, 1.81); apoptotic process (114, 1.86)	10.04
	regulation of cell communication (178, 1.68); regulation of signaling (180, 1.67); regulation of signal transduction (164, 1.72)	9.95
	cell migration (84, 2.03); locomotion (100, 1.87)	7.37
	regulation of multicellular organismal development (106, 1.76); regulation of cell differentiation (97, 1.79); positive regulation of developmental process (70, 1.84)	6.89
	cell adhesion (103, 1.73)	6.23
	cell development (132, 1.92); nervous system development (128, 1.68)	5.5
	cell junction (85, 1.65); focal adhesion (34, 2.33)	4.69
	cardiovascular system development (64, 1.93); circulatory system development (64, 1.93)	4.54
	MAPK cascade (55, 1.87)	4.48
	actin filament-based process (48, 2.04); cytoskeleton organization (67, 1.67)	4.39
	transmembrane receptor protein serine/threonine kinase signaling pathway (30, 2.66); response to transforming growth factor beta (20, 2.66); cellular response to transforming growth factor beta stimulus (19, 2.55)	3.72
	embryo development (65, 1.95); embryo development ending in birth or egg hatching (39, 1.91); chordate embryonic development (38, 1.88)	3.71
	regulation of cytokine production (38, 1.88)	3.19
	apoptotic signaling pathway (39, 1.91); regulation of apoptotic signaling pathway (29, 2.18)	2.7
	positive regulation of metabolic process (143, 1.34); positive regulation of macromolecule metabolic process (134, 1.34); positive regulation of cellular metabolic process (133, 1.34); regulation of transcription from RNA polymerase II promoter (92, 1.43)	2.63
	regulation of protein transport (50, 1.91); regulation of transport (90, 1.43)	2.38

^a^ Values within parentheses indicate the number of genes and fold-enrichment of the functional terms, respectively

^b^ Geometric mean (in –long10 scale) of member’s *P*-values of the corresponding annotation cluster

**Fig. 4:**
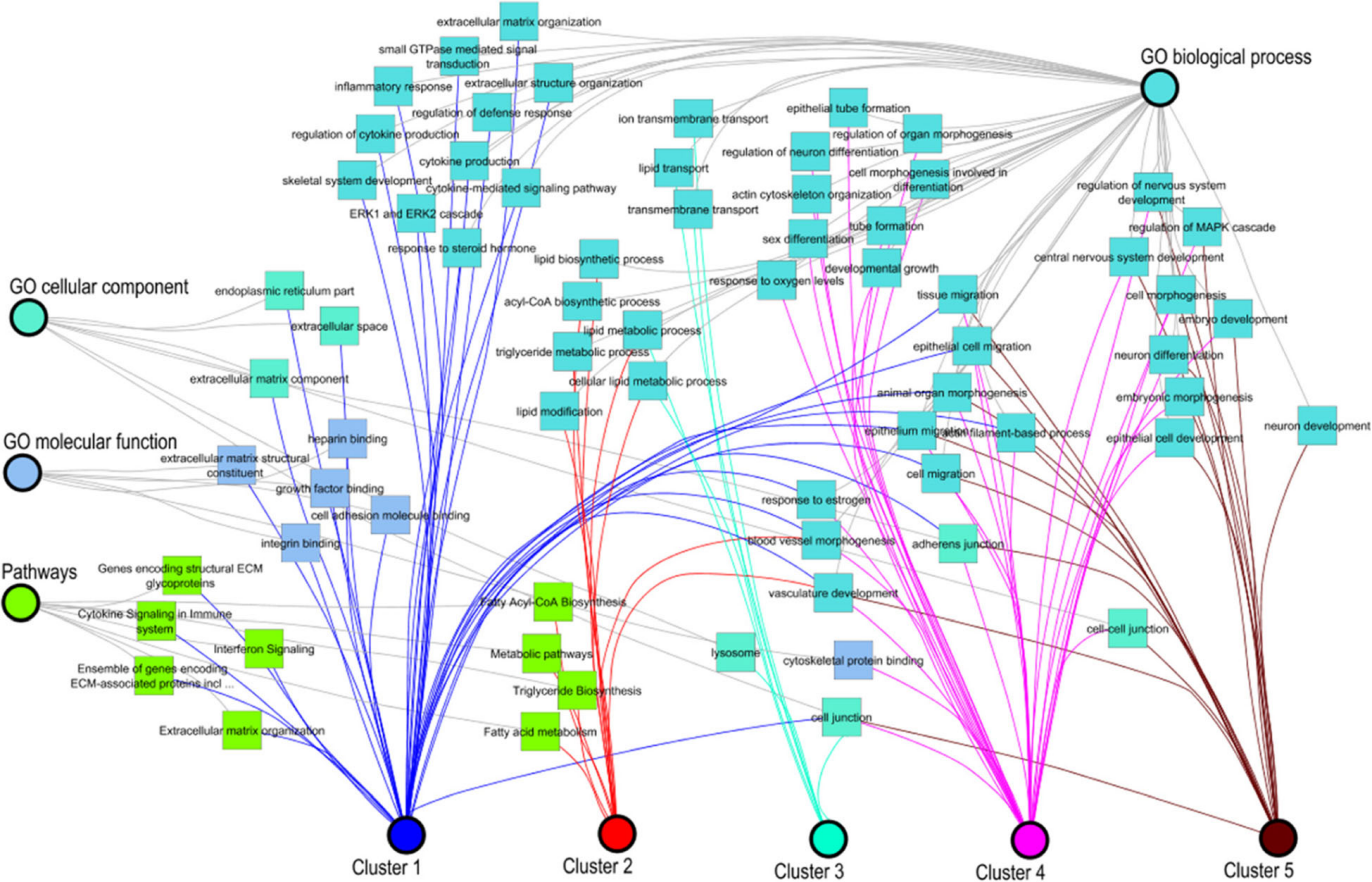
Gene Ontology (GO) functional classification network of clusters. All significant differential genes (human Entrez Gene IDs) from clusters were used as input for the ToppCluster. The following databases were used, i.e. “biological process”, “cellular component”, “molecular function” and pathway. Finally, the data were uploaded in Cytoscape 3.6.0 to modify the network. Nodes were colored based on specificity: red nodes specific for different cluster; nodes for the three GO functions and pathway were in different colors

An overview of a network of enriched functional classification categories for the obtained DEG of the SOTA clusters is shown in Fig. 4. The DEG from these five clusters were analyzed with the online tool ToppCluster for Gene Ontology (GO) and pathway analysis. The most overrepresented categories including response to estrogen, cell migration, actin filament-based process, vasculature development, as well as animal organ morphogenesis were shared in most of the SOTA expression profile clusters (Fig. 4). In addition, there were also functional categories specifically overrepresented in cluster 1, e.g., interferon signaling, extracellular matrix organization, cytokine signaling in immune system, heparin binding, growth factor binding, and cell adhesion molecule binding. Cluster 3 was enriched for transport and cluster 4 in actin cytoskeleton organization, developmental growth, epithelial tube formation, and cytoskeletal protein binding. In addition, neuron development was specifically enriched in cluster 5.

### Top 5% functional annotations of upregulated genes from the embryos on Day 12

To explore more details of the upregulated genes on Day 12, our data were subjected to the online tool DAVID (Gene Ontology (GO) categories and KEGG pathways) to show the systematic functional classification for upregulated genes in the embryos from Day 12 compared with Day 10. The functional categories were selected based on the condition of FDR < 1%, then filtered by a score combining FDR and fold enrichment. The overrepresented GO terms and pathways with the top 5% scores were selected and the results were shown in Table 2. The categories related to ‘morphogenesis’ and ‘development’ were disclosed to be the main functions on Day 12.

**Table 2:** Top 5% functional annotation of upregulated genes of the embryos on Day 12

Category description	Gene Counts	Fold Enrichment	FDR	*P* Value
Prostatic bud formation	7	7.82	0.12	0.0001
Metalloendopeptidase inhibitor activity	10	6.21	0.01	0.0000
Chemorepellent activity	14	5.15	0.00	0.0000
Lateral sprouting from an epithelium	8	6.70	0.10	0.0000
Collagen-activated signaling pathway	6	7.54	0.84	0.0004
Glomerulus vasculature development	12	5.24	0.01	0.0000
Laminin complex	6	7.16	0.82	0.0005
Cardiac septum morphogenesis	25	3.99	0.00	0.0000
Positive regulation of extracellular matrix organization	10	5.58	0.04	0.0000
Negative chemotaxis	17	4.17	0.00	0.0000
Morphogenesis of an epithelial bud	8	6.18	0.19	0.0001
Glomerulus development	24	3.83	0.00	0.0000
Creatine metabolic process	7	6.39	0.57	0.0003
Cardiac septum development	34	3.56	0.00	0.0000
Positive regulation of epithelial cell migration	37	3.54	0.00	0.0000
Ventricular septum development	22	3.51	0.00	0.0000
Renal system vasculature development	12	4.82	0.02	0.0000
Kidney vasculature development	12	4.82	0.02	0.0000
Kidney morphogenesis	32	3.42	0.00	0.0000
Basement membrane	34	3.42	0.00	0.0000
Nephron development	46	3.35	0.00	0.0000
Cardiac chamber morphogenesis	39	3.32	0.00	0.0000
Renal tubule development	31	3.28	0.00	0.0000
Nephron tubule development	30	3.28	0.00	0.0000
Renal tubule morphogenesis	25	3.26	0.00	0.0000
Nephron morphogenesis	25	3.22	0.00	0.0000
Embryonic skeletal joint development	8	5.74	0.35	0.0002
Positive regulation of ossification	27	3.19	0.00	0.0000
Mesonephros development	32	3.12	0.00	0.0000
Smooth muscle cell migration	20	3.53	0.00	0.0000
Ureteric bud development	30	3.11	0.00	0.0000
Extracellular matrix disassembly	27	3.08	0.00	0.0000
Mesonephric epithelium development	30	3.08	0.00	0.0000
Mesonephric tubule development	30	3.08	0.00	0.0000
Cardiac chamber development	47	3.05	0.00	0.0000
Kidney epithelium development	43	3.02	0.00	0.0000
Nephron epithelium morphogenesis	24	3.17	0.00	0.0000

### Signaling pathways related to maternal recognition of pregnancy on Day 12

**Fig. 5:**
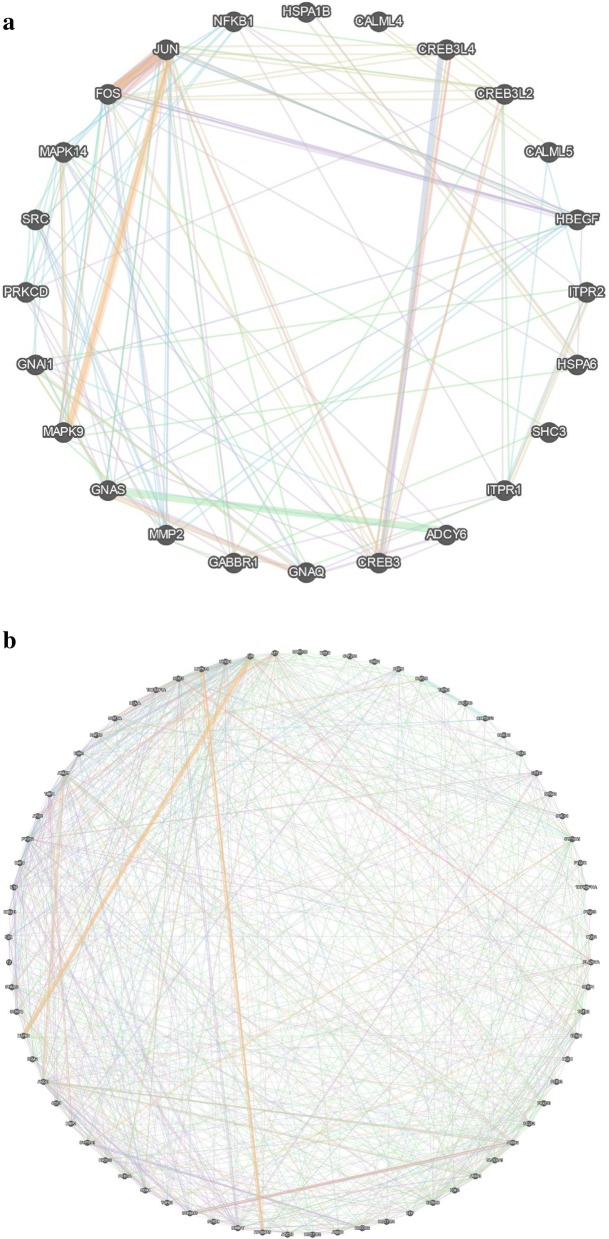
The networks of DEGs involved in estrogen (**a**) and prostaglandin (**b**) signaling pathways. The sources of co-expression, physical interactions, co-localization, pathway, shared protein domains, and genetic interactions were selected to weight the network. One edge indicates one source, and one node means one gene

In the embryos of Day 12, a number of 164 DEGs assigned to a selection of particularly interesting pathways and processes, e.g., estrogen signaling, steroid hormone biosynthesis, prostaglandin (PG) metabolism, signaling and transport are shown in Additional file 3: Table S3. A total number of 24 DEGs including 16 up- and 8 down-regulated genes were found in the estrogen signaling pathways. For PG synthesis, regulation, and transporting, 73 gene were identified as DE (including 47 upregulated and 26 downregulated genes) on Day 12. The networks of these DEGs involved in estrogen and PG signaling pathways are shown in Fig. 5a and b, respectively.

### Comparison of DEGs in the elongating conceptus between pig and sheep

**Fig. 6:**
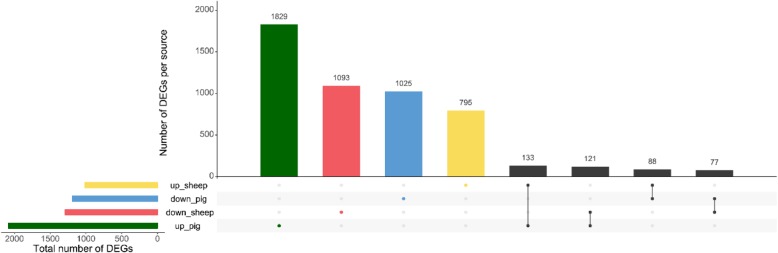
Upset plots illustrating the number of differentially expressed genes in porcine and ovine embryos (B). Green and blue indicate the up- and down-regulated genes in porcine embryos. Yellow and red indicates the up- and dwon-regulated genes in ovine embryos. The green, red, blue, and yellow points indicates the genes were specifically expressed in one species, while the black plots mean these genes are commonly expressed in two species

Data of dynamic transcriptome changes from ovoid to a filamentous conceptus in sheep [22] have been adopted to compare with our findings (from Day 10 to Day 12 in pigs, and from Day 12 to Day 14 in sheep). The results show that the majority of the DEGs (1829) were identified as upregulated specifically in porcine embryos (Fig. 6), and 1025 gene were identified as downregulated. A number of 1093 downregulated genes and 795 upregulated genes were specifically expressed in sheep embryos. Besides, 133 upregulated and 77 downregulated genes were commonly expressed during the elongation in both species, while 209 genes had different regulations in pig and sheep embryos. The detailed information of these DEGs is depicted in Additional file 4: Table S4.

### Analysis of expression of genes located on the X chromosome in the three embryo stages

**Fig. 7:**
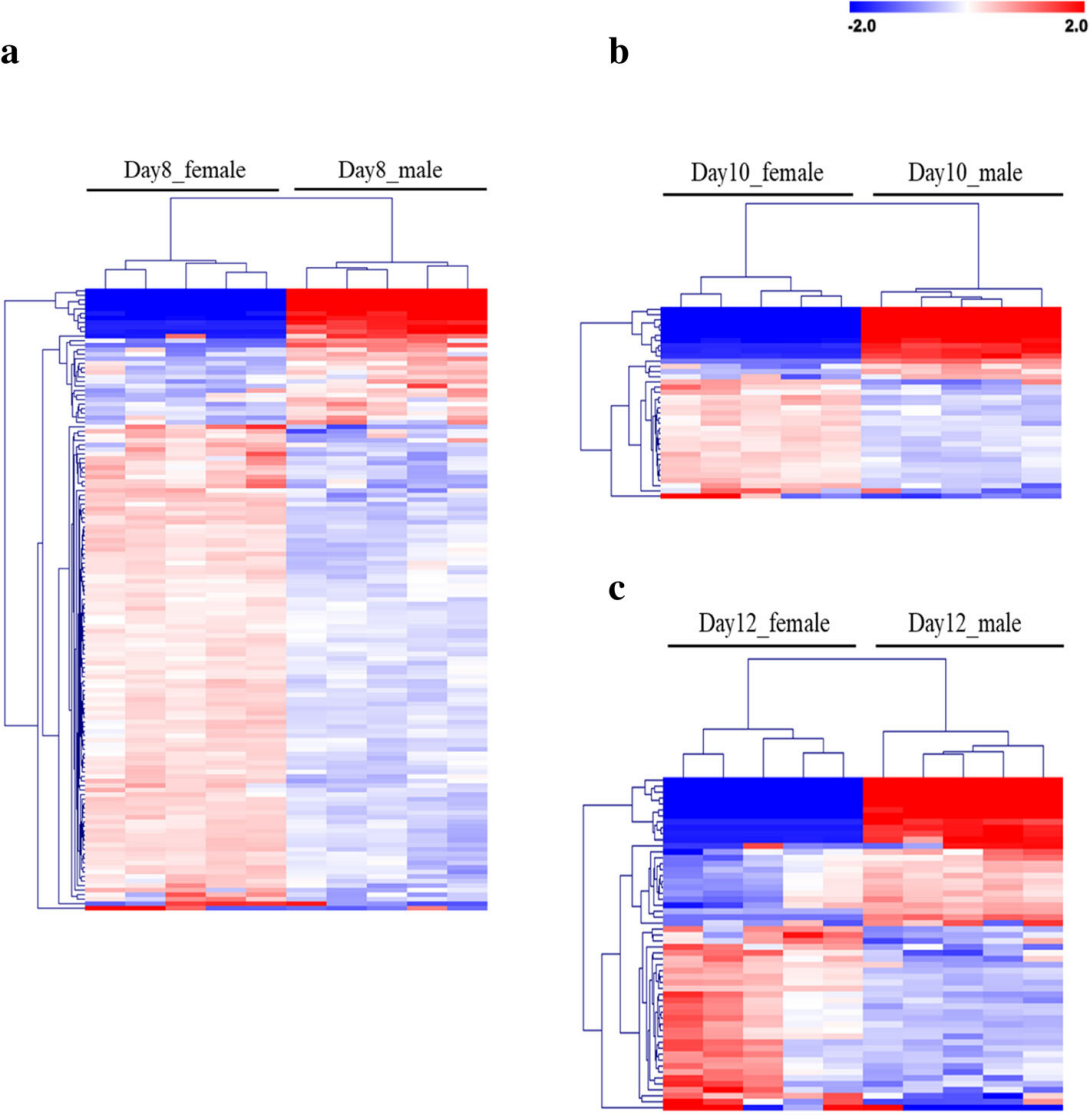
Hierarchical cluster analysis of differentially expressed genes between female and male embryos identified for the (**a**) Day 8, (**b**) Day 10, and (**c**) Day 12 embryos. Mean-centered expression values (log2 counts per million of sample – mean of log2 counts per million of all samples) for the female and male embryos are shown with significant differences in gene expression (FDR < 5%). Color scale is from − 2 (blue, lower than mean) to 2 (red, higher than mean). Each row represents one gene, each column represents one embryo

**Fig. 8:**
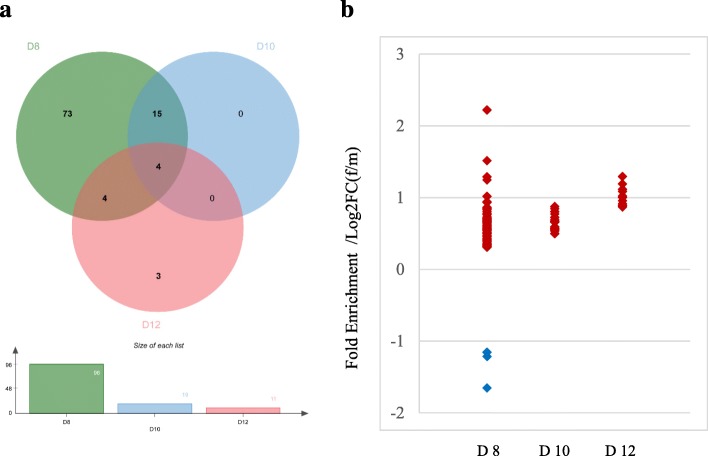
Venn diagram for X-linked differentially expressed genes (**a**) and expression profile of X-linked differentially expressed genes (**b**). Green, blue, and pink filed in Venn graph indicate X-linked differentially expressed genes on Day 8, 10, and 12 of the embryos, respectively. The red and blue plots in the scatter mean the up and down-regulated genes on the X chromosome in female embryos, respectively

At an FDR of 5%, a number of 137, 37, and 56 differentially DEG between female and male embryos were obtained on Days 8, 10, and 12, respectively. A hierarchical cluster analysis of these DEG is shown in Fig. 7a, b, and c. With respect to higher or lower expression of DEG in female compared to male embryos, 107 upregulated and 30 downregulated, 23 upregulated and 14 downregulated, and 31 upregulated and 25 downregulated genes were identified on Days 8, 10, and 12, respectively (Additional file 5: Table S5). Regarding chromosomal location of these DEG, a number of 96, 19, and 11 X-linked DEG were found on Days 8, 10, and 12, respectively (Fig. 8a). The number of DEG located on the X chromosome decreased on Day 12 compared with Day 8 embryos (20% vs 70% of the DEG). Four X-linked genes, namely B-cell receptor-associated protein 31 (*BCAP31*), family with sequence similarity 3 member A (*FAM3A*), ribosomal protein L10 (*RPL10*), and tafazzin (*TAZ*), were upregulated in female embryos on all 3 days (Additional file 5: Table S5). All DEG located on the X chromosome were upregulated except three genes, namely family with sequence similarity 155 member B (*FAM155B*), collagen type IV alpha 5 chain (*COL4A5*) and spindlin-2B (*LOC100526148*), that were downregulated in female embryos on Day 8 (Fig. 8b). More detailed information about the DEG between female and male embryos for each stage is shown in Additional file 5: Table S5.

### Functional annotations for sex-based DEGs and X-linked DEGs in the embryos

The functional classification based on the DEGs from the comparison between female and male embryos was identified at each stage (Table 3). A number of 22 functional categories were identified on Day 8. Among these categories, genes involved in “phosphatidylinositol-3,5-bisphosphate 3-phosphatase activity” had the highest enrichment. Interestingly, on Days 10 and 12, only one category, the “translational initiation”, was enriched in both two stages.

**Table 3:** Overrepresnted functional categories for the DEGs between female and male embryos

Stage	Category Description	Gene Counts	Fold Enrichment	FDR	*P* Value
Day 8	intracellular ribonucleoprotein complex	18	3.26	0.0375	0.0000
	ribonucleoprotein complex	18	3.26	0.0381	0.0000
	cytosol	43	1.80	0.0503	0.0000
	oxidation-reduction process	19	2.87	0.1475	0.0001
	RNA binding	25	2.29	0.1836	0.0001
	poly(A) RNA binding	20	2.54	0.3377	0.0002
	translational initiation	8	6.29	0.4787	0.0003
	ncRNA processing	11	3.98	0.7393	0.0004
	oxidoreductase activity, acting on the CH-OH group of donors, NAD or NADP as acceptor	6	7.78	1.4327	0.0010
	cytosolic part	8	4.77	1.9001	0.0014
	ribonucleoprotein complex biogenesis	11	3.41	2.4027	0.0014
	cytosolic ribosome	6	6.84	2.4425	0.0018
	peptide biosynthetic process	13	2.90	2.8247	0.0016
	oxidoreductase activity, acting on CH-OH group of donors	6	6.65	2.8642	0.0020
	ribosome biogenesis	9	3.99	3.1336	0.0018
	phosphatidylinositol-3,5-bisphosphate 3-phosphatase activity	3	41.39	3.1490	0.0022
	nucleotide binding	28	1.78	3.3566	0.0024
	nucleoside phosphate binding	28	1.78	3.3768	0.0024
	cell growth	10	3.41	4.3966	0.0026
	mitochondrial part	16	2.30	4.5972	0.0034
	cellular macromolecular complex assembly	16	2.37	4.6380	0.0027
	RNA processing	15	2.45	4.9544	0.0029
Day 10	translational initiation	4	15.32	3.0113	0.0019
Day 12	translational initiation	5	11.90	1.1225	0.0007

X-linked DEGs were analyzed to reveal the related functional categories (Table 4). The majority of functions were similar as the categories from the sex-based DEGs on Day 8. However, no functional categories with significant fold enrichment from the X-linked genes were identified on days 10 and 12.

**Table 4:** Overrepresnted functional categories for the X-linked DEGs between female and male embryos on Day 8

Category Description	Gene Counts	Fold Enrichment	FDR	*P* Value
Poly(A) RNA binding	18	2.99	0.0894	0.0001
ncRNA processing	11	5.06	0.0954	0.0001
RNA binding	20	2.40	0.5231	0.0004
Intracellular ribonucleoprotein complex	14	3.12	0.5849	0.0004
Ribonucleoprotein complex	14	3.11	0.5920	0.0004
Ribosome biogenesis	9	5.08	0.6241	0.0004
ncRNA metabolic process	11	3.65	1.3126	0.0008
RNA processing	14	2.91	1.4558	0.0008
Cytosol	33	1.70	1.4924	0.0011
Ribonucleoprotein complex biogenesis	10	3.94	1.5288	0.0009
Mitochondrial part	15	2.65	1.6200	0.0012
Phosphatidylinositol-3,5-bisphosphate 3-phosphatase activity	3	54.12	1.8057	0.0013
Chromosome organization	16	2.56	1.9363	0.0011
Mitochondrial membrane	12	3.04	2.2622	0.0017
Oxidation-reduction process	14	2.69	2.9103	0.0017
Phosphatidylinositol-3-phosphatase activity	3	42.52	2.9429	0.0021
Phosphatidylinositol monophosphate phosphatase activity	3	42.52	2.9429	0.0021
Cytosolic part	7	5.13	3.0904	0.0023
Mitochondrial envelope	12	2.87	3.5318	0.0026
Mitochondrion organization	11	3.19	3.5913	0.0021
Anatomical structure homeostasis	8	4.30	4.1306	0.0024
rRNA processing	7	4.92	4.8004	0.0028

## Discussion

Prior to attaching to the maternal uterine epithelial surface, porcine embryos normally undergo programmed differentiation and expansion. This reflects the rapid morphological changes between Days 8 and 12, which results from various hormonal and gene expression changes. The transcriptome of the embryo either in autosomal or sex chromosomes is hypothesized to be dynamic, but only little evidence supports this hypothesis. By using RNA-Seq, the present study analyzed embryos sampled at three critical time points and revealed a range of phase-specific gene expressions regulating embryo elongation, development and communication with the maternal uterus. These events are critical for successful implantation in pigs.

### Genes related to conceptuses elongation (cell growth, cell movement, and cellular remolding)

The current results revealed that the elongating conceptuses under investigation displayed various dynamic gene expression changes most highly expressed on Day 12, especially relating to cell growth, cell movement as well as cellular remolding.

A number of growth factors were found to have increased gene expression on Day 12, including insulin growth factor (*IGF*) family, fibroblast growth factor (*FGF*) family, transforming growth factor beta 1 (*TGFβ1*), transforming growth factor beta 2 (*TGFβ2*), platelet derived growth factor receptor alpha (*PDGFRA*), insulin like growth factor binding protein 5 (*IGFBP5*), and fibroblast growth factor receptor 2 (*FGFR2*). These results confirm a related study in mouse, where a range of growth factors were produced by the preimplantation embryo and the reproductive tract, and many of the respective receptors were detected on the embryo surface [23]. The *TGFβ* was regarded as an essential factor in modulating extracellular matrix (ECM) protein expression and the accumulation of ECM proteins results in increasing cell migration [24]. Expression of *TGFβ1* and *TGFβ3* were observed to be unregulated in porcine conceptuses on Day 10 compared with Day 8, and mRNA of *TGFβ1*, *TGFβ2*, and *TGFβI* were still increasing except *TGFβ3* was decreasing from Day 10 to Day 12. From our findings, the increasing *TGFβ* may contribute to some extent to morphological changes of the embryo via migrating cells and remolding tissues. Growth differentiation factor 6 (*GDF6*) was observed with the highest expression on Day 12. Recently, *GDF6* gene was detected in several distinct embryonic locations in mouse, which facilitates the skeletal and soft tissues formation [25]. We found placenta expressed transcript 1 (*PLET1*) upregulated on Day 12, which is in line with the previous result that *PLET1* was found with high expression in elongated conceptuses [26], indicating a strong activity of trophoblast differentiation [27].

Coordination between assembly and disassembly of actin filaments is a key factor to provide a driving force that initiates the cell movement in animals [28]. It is reported that the cellular motility in vitro can be reconstituted by a core set proteins including actin, actin related protein2/3 (Arp2/3) complex, actin depolymerizing factor (ADF)/cofilin, profilin, and capping protein [29]. Consistently, the current study observed that genes coding actin proteins, actin binding LIM protein 1 (ABLIM1), actin related protein 2/3 complex subunit 1B (ARPC1B), cofilin 2 (CFL2), profilin 1 (PFN1), and capping actin protein gelsolin like (CAPG) were differently expressed from Days 8 to 12. With reference to porcine embryos, the filamentous actin cytoskeleton reassembly is rather outstanding in the early pregnant stage implying that compaction and blastogenesis are activated during the preimplantation period [30]. Herein, we report that the gene coding for cytoskeleton associated protein 4 (CKAP4) revealed highest expression on Day 12 suggesting that stage-specific changes in actin organization occur between Days 8 and 12 of pregnancy. This is consistent with the formation of cytoskeletal elements occurring during mouse embryo preimplantation stage [31]. On the other hand, the low expression of genes coding for two major cytoskeletal proteins (desmin (*DES*) and vimentin (*VIM*)) was observed. The expression of *VIM* was greater in Day 12 conceptuses compared to Day 8 and 10 conceptuses, which is in line with previously observed VIM protein patterns indicating that mesodermal differentiation and migration activities are greater during the filamentous stage [32–34]. Though DES and VIM proteins were confirmed to be expressed dynamically, their transcripts were kept a low level based on the present results. Though DES and VIM regulating filaments were found not to be essential for embryo development, the cytokeratin formation is an initial step for the epithelium differentiation [31]. A *DES* and *VIM* co-expression may exist to make up the insufficient filament protein production in developing embryonic tissue [35].

The majority of genes regulating the cell differentiation, growth and movement were expressed highly on Day 12. As indicated in Fig. 2b, the total number of activated genes increased gradually along developmental progression suggesting that Day 12 of pregnancy is a critical period with more pathways and functions involved in embryo elongation. Meanwhile, the expressions of some genes reached the peak on Day 10, like *KRT*, *MDK* that are responsible for blastocyst formation [36] and embryo survival [37].

### Genes related to embryo development

It is known that the transformation of morphology is essential for embryo survival as the increased placental surface area can improve the nutrient exchange between the conceptus and the uterus [2]. Lipids are not only components of cellular membrane and cytoplasm but also of great importance in producing energy that is essential for proper embryo development [38]. Genes related with lipid biosynthetic process, such as lipoprotein (*LPL*) and acyl-CoA synthetase long chain family member 3 (*ACSL3*), were found to be expressed in the present study, especially fatty acid binding protein 3 (*FABP3*) and ELOVL fatty acid elongase 6 (*ELOVL6*), which reached a peak on Day 10. As reported previously, the content of lipids in pig embryos was markedly decreased in the late blastocyst stage [38], which proves that the energy for embryo development from Day 8 to Day 10 is provided by lipid synthesis and metabolism. As the embryo achieves a more advanced development from Day 10 onwards, genes involved in the category of energy production were rationally downregulated on Day 12.

Significant different gene expression of *KRT8* and *KRT18* were found between bovine morula and blastocyst embryos indicating their roles in blastocyst formation and embryo implantation [39]. The latter, KRT18 mRNA and protein were confirmed to be differentially expressed in bovine embryo development and it was regarded as a marker for blastocyst formation [36]. The present findings were in line with a previous report that expression of *KRT18* mRNA was decreased in the filamentous conceptus compared with the ovoid conceptus, implying that *KRTs* play a role in trophectoderm development [40]. The neurite growth-promoting factor, midkine (MDK) protein is a developmentally regulated heparin binding cytokine that is induced by retinoic acid [41, 42]. The *MDK* transcripts reached at a peak in the Day 10 conceptus of our study suggesting that it may be associated with extra-embryonic tissue development from Day 8 to Day 10 [40] rather than the tissue remodeling from Day 10 to Day 12. The genes under similar regulations between porcine and ovine elongating embryos (as shown in Fig. 6), revealing the conserved DEGs in two species. While the opposite regulated genes from these two species may likely indicate the distinct pathways that facilitate embryo elongation in pig different from sheep.

The genes involved in nutrient requirements displayed stage-specific expression. For example, Day 10 embryos required more energy reflected by the high gene expression coding for lipid and energy synthesis. In addition, novel and stage-specific genes transcripts were discovered regarding estrogen and embryo development.

### Genes related to embryo-maternal interaction

Seven integrin alpha genes (*ITGA2*, *ITGA3*, *ITGA5*, *ITGA6*, *ITGA9*, *ITGA10*, and *ITGAL*) and five integrin beta genes (*ITGB2*, *ITGB3*, *ITGB5*, *ITGB6*, and *ITGB8*) were notably differentially expressed. A higher abundance of *ITGA3*, *ITGB6*, and *ITGB8* was identified on Day 10 compared with Day 8. However, expression level of nine integrin genes (including *ITGA2*, *ITGA3*, *ITGA6*, *ITGA10*, *ITGAL*, *ITGB2*, *ITGB3*, *ITGB5*, and *ITGB6*) higher abundant at Day 12 compared to Day 10. Integrins have been recognized as cell surface adhesion receptors [43]. According to the network of ligand receptor-mediated multicellular signaling from the FANTOM5 database of human cells [44], the ligand of integrin has been located in the endometrium on Day 12 of pregnancy in pigs [45, 46]. The expression of integrins with the detection of their ligands indicates the role of cell adhesion taking place in the maternal-embryo crosstalk, especially on Day 12, a time with an intensive embryo-maternal interaction.

A number of genes coding gap junction protein (GJA1, GJB1, GJB3, GJB5) were highly expressed, especially on Day 12. Gap junction is a complex of proteins locating distinctly on the plasma membrane of bordering cells to establish the intercellular connection [47]. The gene coding desmosome associated protein, *PNN*, was likewise among the highly expressed transcripts in the present study. Desmosomes were reduced in the mouse uterine epithelium during the preimplantation revealing that the reduction in desmosome adhesion contributes to embryonic-penetration in luminal epithelium [48]. In contrast to the invasive implantation in mouse, a high expression of *PNN* was observed in all three stages of development under investigation, which leads us to assume that there was no penetrating process due to the specific non-invasive implantation in pigs. In our study, genes coding tight junction proteins (TJP1, TJP2), and *TJP3* were upregulated, which are produced by embryonic cells during preimplantation period and act as a tight junction for polarized transporting, and intercellular integrity and signaling [49].

The expression of *IL1B* in the embryo during the preimplantation was reported with dynamic changes, however, the pattern currently seems to be controversial. Ross et al. reported that the expression of *IL1B* was increasing from Day 10 to Day 12 along with the embryo elongation [12]. Another related study showed that the level of *IL1B* mRNA of porcine conceptus was decreased on Day 12 when compared with Day 11 [50]. Similar to the later, we noticed that transcripts of *IL1B* mRNA reached a peak on Day 10 compared to Days 8 and 12. It has been shown that IL1 was induced by estrogen and progesterone, while high levels of estrogen and progesterone could inversely inhibit IL1 secretion [51, 52]. Therefore, we propose that from Days 8 to 10, the increasing secretion of estrogen by the elongating conceptuses most likely stimulates *IL1B* expression. However, the large amount of accumulated estrogen on Day 12 thereafter inhibit *ILIB* transcription. *IL1B* has a unique form in pig conceptuses, namely *IL1B2,* which was highly expressed on Day 10. Though interleukin 1 receptor type 1 (*IL1R1*) and interleukin 1 receptor accessory protein (*IL1RAP*), receptors for the IL1B-signaling system, were found low expression from Day 8 to 12. The *IL1B* of the embryo together with *IL1R1* and *IL1RAP* initiate the IL1B-signaling system plays an important role in implantation by regulating expression of endometrial genes for prostaglandin (PG) synthesis [53].

Along with morphological changes, expression patterns indicate the intimate dialogue between endometrium and embryo. Over time, the communication intensity increases indicated by the increased expressions of various genes involved in the cell surface adhesion and gap junction.

### The maternal recognition of pregnancy on Day 12

The rapidly elongated embryo secretes large amount of estrogen on Day 12, which initiates the maternal recognition of pregnancy in pigs. Expression of estrogen receptor has been described in the endometrium of sows during early pregnancy [54]. Cytochrome P450 family members are involved in estrogen synthesis and metabolism [55, 56]. In the current study, a number of genes coding specific CYP members, i.e. CYP11A1, CYP17A1, CYP19A1, CYP2C9, CYP2C18, CYP2S1, and CYP4F22 were identified as DEGs specifically on Day 12. Hydroxysteroid 17-beta dehydrogenase 1 (*HSD17B1*) catalyzes cholesterol to estrogen in endometriosis [57], and is massively expressed in the trophectoderm rather than in the embryonic disc of the porcine conceptus tissue on the day 12 [58]. Compared with Day 10, the expression of *HSD17B1* was decreased on Day 12, suggesting that HSD17B1 secreted from the trophectoderm was not the most important component in the estrogen synthesis. Phospholipase A2 family members (*PLA2s*), the key enzymes for the release of PG precursor molecules [59], were upregulated in the embryos on Day 12. In contrast, endometrial *PLA2s* were described as downregulated on Day 12 in pigs [46]. The differential expression of *PLA2s* in embryos and endometrial cells point towards the regulation of the corpus luteum maintenance via PG. The transporter of PGs, the solute carrier organic anion transport family member 2A1 (*SLCO2A1*) mRNA was upregulated in the embryos on Day 12 compared with Day 10. The large amount of estrogen secreted from the embryos could stimulate the endometrium to switch the secretion of PG from endocrine to exocrine. Taken together, the genes involved in estrogen secretion and regulation of PG synthesizing, metabolizing and transporting proteins revealed a complex pattern of maternal recognition of pregnancy.

### DEGs between female and male

Dosage compensation for X-linked genes occurs by inactivating one X chromosome in the female during embryonic development, the event of which is highly conserved in the mammal and essential for embryogenesis [60]. The current findings revealed a number of differentially expressed genes between female and male pig embryos, namely 137, 37, and 56 on Day 8, 10, and 12, respectively. Distributing the gene location on the chromosomes, 100, 19, and 11 genes were X-linked DEG on the respective days. In bovine blastocysts, more DEG, namely 382 genes, were detected between female and male embryos, from which 218 genes were X-linked [21]. In previous study, around 600 DEG were discovered in between female versus male mouse blastocysts using transgenesis and microarray analyses [61]. Thus, less X-linked genes were detected in pigs than mouse and cows. Sex chromatin as a part of the X chromosome was first detected at the blastocyst stage with 45 cells in porcine embryos, which was an approximate guide to the presence of an inactive chromosome [62]. This could also be a reason that XCI started partially before Day 8 in porcine embryos. Additionally, the majority of the upregulated genes in female embryos were X-linked on Day 8, similar to the results observed in bovine [21]. Despite that parts of the X-linked genes were not differentially expressed before Day 8, our results still provide the evidence that 81 X-linked gene expression are changed between male and female embryos from Day 8 to Day 10, and eight from Day 10 to Day 12. Therefore, the data allow the assumption that XCI probably proceeds gradually and causes the decreasing of X-linked DEG from Day 8 to Day 12.

Prior to the initiation of XCI, all X-linked genes should be present as 2-fold dose in females [63]. In fact, most X-linked genes determined here were less than two-fold expressed in female embryos on Day 8 compared to males. The housekeeping X-linked gene glucose-6-phosphate dehydrogenase (*G6PD*) has been considered as a potential candidate involved in sex difference [64]. In our findings, *G6PD* transcripts were high abundant, but only 1.5-fold in female embryos on Day 8 compared to males. Moreover, *G6PD* was not differentially expressed on Days 10 and 12. Previous studies reported that inactivation of paternal X-linked genes was often incomplete, which is associated with the specific locus, species, and tissue, respectively [65, 66]. In addition, it is known for many years that sex chromatin and late replication of a X chromosome do not occur in all cells of the embryo at the same time [67]. This is likely the reason for most genes on X chromosomes being differentially expressed on Day 8 and no difference observed on Days 10 and 12. Functional annotations for the sex-based DEGs and X-linked DEGs revealed that the majority of functional categories were attributed by the DEGs on the X chromosome rather than the autosome on Day 8. Neither on Day 10 nor Day 12, significant functional categories were detected, indicating that the compensation on the X chromosome has balanced the difference between female and male embryos. Overall, based on the comparison of X-linked genes between female and male preimplantation embryos, the number of DEG on X chromosome were decreasing along with the progression of pregnancy. These finding suggested that the compensation between female and male has occurred before Day 8, and would persist until Day 12 with most X-linked genes expressed with similarity between male and female embryos.

## Conclusion

In conclusion, we identified comprehensive transcriptome changes associated with embryo elongation, development, and embryo-maternal interaction during the preimplantation period. A number of biological processes and pathways with temporal changes were revealed governing the embryonic cell movement and remodeling to form the elongated embryo. Genes involved in cell communication and adhesion were highly expressed on Day 12, which indicates the increased interaction between the mother and embryo. Bioinformatics analyses of gene expression between female and male embryos showed that a number of X-linked genes were differentially expressed on Day 8. These DEGs disappeared gradually along with the embryo elongation on Days 10 and 12. The latter findings suggest that dynamic changes of transcriptome on the X chromosome may reveal the changed dosage compensation between sexes before embryo implantation in pigs.

## Methods

The animal experiment was conducted in Freising, Germany and approved by the District Government of Upper Bavaria and were in accordance with the accepted standards of humane animal care in Germany.

### Animal experiment

The animals were owned and kept at the Research station “Thalhausen” of the Technical University of Munich, Freising, Germany. Twelve German Landrace × Pietrain crossbred gilts were cycle synchronized using Altrenogest ReguMate® for twelve days, then Intergonan® (PMSG) at 750 iU was applied once in the following evening, and Ovogest® (human chorion gonadotropin) at 750 iU was applied once 3.5 days later. The next day (day 0), all animals were inseminated with sperm of the same single Pietrain boar. The animals were slaughtered in a commercial slaughterhouse. Four gilts were slaughtered by stabbing in the neck for bloodletting after anesthesia each on Days 8, 10, and 12 post insemination, respectively. The respective gilts were randomly assigned to the date of slaughtering. The reproductive tracts were collected immediately after slaughter.

### Embryo collection

The complete embryos (including embryonic disc and trophectoderm cells) were flushed from the uterus using 10 ml of phosphate-buffered saline (PBS, pH 7.4) per horn, and then were transferred into a petri-dish containing PBS for morphological observation. All embryos were then individually shock-frozen in liquid nitrogen immediately and stored at − 80 °C for further DNA and RNA analyses.

### DNA sex determination and RNA-seq library preparation

All embryos underwent total DNA and RNA extraction using the AllPrep DNA/RNA Micro Kit (Qiagen, Valencia, CA, USA) according to the manufacturer’s recommendation. The integrity and the quantity of the RNA were assessed on the Agilent 2100 Bioanalyzer (Agilent Technologies, Waldbronn, Germany). The DNA samples were used for sex determination by measuring relative gene expression with quantitative real-time PCR (qPCR). Gene-specific primers (SRY and Histone genes) were designed with online tool NCBI Primer-BLAST (see Additional file 6: Table S6). The amplification products were obtained from qPCR with reaction mixture including 2 × FastStart Universal SYBR Green Master 10 μl, DNA 1 μl, forward primer 0.6 μl, reverse primer 0.6 μl, and add water to 20 μl. The following qPCR program: 95 °C for 10 min, amplification for 40 cycles at 95 °C for 15 s and 60 °C for 60 s, and ∆Ct value method was used for relative quantification.

We then randomly selected 5 embryos per time point and sex of high RNA quality (RIN > 8) for RNA-seq. After RNA extraction, 100 ng total RNA was used as input for the Illumina TruSeq Stranded mRNA library construction (Illumina Inc., San Diego, CA, USA) regarding the manufacturer’s recommendation. Briefly, RNA was fragmented and random primers were hybridized for cDNA synthesis. The resulting cDNA was followed by 3′ adenylation and adapter ligation. Finally, PCR amplification was performed with the subsequent protocol (98 °C for 30 s; 15 cycles of 98 °C for 10 s, 60 °C for 30 s, 72 °C for 30 s; 72 °C for 5 min). Library quality control was performed with Agilent 2100 Bioanalyzer (Agilent Technologies, Waldbronn, Germany), followed by sample pooling. Finally, pooled libraries was sequenced as 100-bp single-end on the HiSeq 2500 (Illumina Inc.) platform.

### RNA sequencing data analysis

Analysis of RNA-seq data was performed using a locally installed version of Galaxy [68]. Raw reads were quality trimmed with Trim Galore and 3 bp were removed from the 5′ end of each read. All sequences were mapped to the *Sus scrofa* genome (version 11.1) from NCBI (ftp://ftp.ncbi.nih.gov/genomes/Sus_scrofa/GFF) and filtered by CPM cutoff. The resulting read count table with CPM was used for statistical analysis in EdgeR to identify differentially expressed genes (DEG) by using GLM robust method [69]. The DEG between the female and male embryos were filtered with a false discovery rate (FDR) at 5%, and DEG between the different stages were filtered with FDR < 0.1% and │log2 fold change│ > 1. Hierarchical cluster analysis was performed for preliminary assessments of the number of gene clusters in MultiExperiment Viewer (MeV) [70], then the list of DEGs from three stages were used for Self Organizing Tree Algorithm (SOTA) clustering. Gene ID of DEGs in each cluster were uploaded into DAVID for gene ontology (GO) and pathway analyses [71]. DEGs from individual clusters were analyzed with the online tool Toppcluster (http://toppcluster.cchmc.org) to generate the overview networks between the GO categories and pathways and the *p*-Value cutoff was set at 0.05 then improved on Cytoscape to show the final results [72]. The network of the DEGs involved in the estrogen and prostaglandin signaling pathway was analyzed on online tool GeneMANIA.

## Additional files

Additional file 1: **Table S1.** DEGs across the stages from porcine embryos on Day 8, 10, and 12, respectively. (XLSX 2707 kb)

Additional file 2: **Table S2.** Functional category analyses for genes differentially expressed in each cluster. (XLSX 665 kb)

Additional file 3: **Table S3.** DEGs involved in signaling pathways of maternal recognition of pregnancy. (XLSX 431 kb)

Additional file 4: **Table S4.** Comparison of DEGs in the elongating embryos between pigs and sheeps. (XLSX 133 kb)

Additional file 5: **Table S5.** DEGs between female and male embryos on Day 8, 10, and 12, respectively. (XLSX 68 kb)

Additional file 6: **Table S6.** Gene-specific primers for SRY and Histone genes. (DOCX 12 kb)

## Data Availability

All date used in this study have been included in the article and its Additional files. The sequence data (GSE113366) is available at National Center for Biotechnology Information (NCBI) Gene Expression Omnibus (https://www.ncbi.nlm.nih.gov/geo/query/acc.cgi?acc=GSE113366).

## Abbreviations

ABLIM1: Actin binding LIM protein 1
ACSL3: Acyl-CoA synthetase long chain family member 3
Arp2/3: Actin related protein2/3
ARPC1B: Actin related protein 2/3 complex subunit 1B
BCAP31: B-cell receptor-associated protein 31
CAPG: Capping actin protein gelsolin like
CFL2: Cofilin 2
CKAP4: Cytoskeleton associated protein 4
COL4A5: Collagen type IV alpha 5 chain
CYP: Cytochrome P450 family members
DEGs: Differentially expressed genes
DES: Desmin
ECM: Extracellular matrix
ELOVL6: ELOVL fatty acid elongase 6
ERK1/2: Extracellular signal-regulated kinase 1/2
FABP3: Fatty acid binding protein 3
FAM155B: Family with sequence similarity 155 member B
FAM3A: Family with sequence similarity 3 member A
FGF: Fibroblast growth factor
FGFR2: Fibroblast growth factor receptor 2
G6PD: Glucose-6-phosphate dehydrogenase
GDF6: Growth differentiation factor 6
GJs: Gap junction protein
GO: Gene Ontology
HSD17B: Hydroxysteroid 17-beta dehydrogenase 1
IGF: Insulin growth factor
IGFBP5: Insulin like growth factor binding protein 5
IL1B: Interleukin one beta
IL1R1: Interleukin 1 receptor
IL1RAP: Interleukin 1 receptor accessory protein
ITGs: Integrin genes
KRTs: Cytokeratins
LPL: Lipoprotein
MAPK: Mitogen activated kinase-like protein
NFKB: Nuclear factors kappa-B
PDGFRA: Platelet derived growth factor receptor alpha
PFN1: Profilin 1
PG: Prostaglandin
PGE: Prostaglandin E2
PGF: Prostaglandin F2α
PGK1: Phosphoglycerate kinase 1
PLET1: Placenta expressed transcript 1
PTGS: PG-endoperoxide synthase
PTGS2: Prostaglandin-endoperoxide synthase 2
RPL10: Ribosomal protein L10
S18: 18S ribosomal RNA
TAZ: Tafazzin
TGFβ: Transforming growth factor beta
TJPs: Tight junction proteins
UBC: Ubiquitin
VIM: Vimentin
XCI: X-chromosome inactivation
