# Supplementary material for: Differential transcriptome dynamics during the onset of conceptus elongation and between female and male porcine embryos
Source: BMC Genomics. 2019 Aug 28;20:679. doi: 10.1186/s12864-019-6044-z (PMC6714402; doi:10.1186/s12864-019-6044-z)
Supplement: Supplementary file 6 — Table S6. Gene-specific primers for SRY and Histone genes. (DOCX 12 kb) [file 12864_2019_6044_MOESM6_ESM.docx]

Gene-specific primers for SRY and Histone genes

| Gene | Primer | |
| --- | --- | --- |
| SRY | forward | GAACGCTTTCATTGTGTGGTC |
|  | reverse | GAAGAATGGGCGCTTTTCGG |
| Histone | forward | AATTAAGACGCAAGTGTTCTGG |
|  | reverse | CCACAGGTTTCCATCATAAGGTC |
